# Results of hearing aids use dispensed by a publicly-funded health service

**DOI:** 10.5935/1808-8694.20130126

**Published:** 2015-10-08

**Authors:** Juliana Harumi Iwahashi, Isabela de Souza Jardim, Ricardo Ferreira Bento

**Affiliations:** a MSc in Sciences, Medical School of the University of São Paulo; Speech and Hearing Therapist; b PhD in Sciences, Medical School of the University of São Paulo; Speech and Hearing Therapist; c Full Professor of Otorhinolaryngology, Medical School of the University of São Paulo; Otorhinolaryngologist

**Keywords:** adult, hearing aids, hearing loss, public health, unified health system

## Abstract

Hearing aid use and satisfaction can be used to analyze the effectiveness of hearing rehabilitation, an issue few explored in Brazilian users.

**Objective:**

Evaluate hearing aid use dispensed in a publicly-funded health service after one year, the interventions needed and users' satisfaction.

**Method:**

Prospective observational cross-sectional study. Hearing aid users were invited by telephone to participate in an evaluation of hearing aid use.

**Results:**

200 hearing aid users attended the evaluation (47% of sample loss); 76.5% of the subjects were using hearing aids bilaterally, 10.5% unilaterally and 13.0% none of them; 99.5% of users needed some kind of intervention. Users who kept wearing their hearing aids were considered “satisfied”.

**Conclusion:**

Active search by telephone showed that hearing aid users who attend this publicly-funded health service have difficulties to attend follow-up appointments. Most of the users that came for evaluation kept using their hearing aids; non-use rate was 13%. Practically all hearing aid users needed some kind of intervention. Despite the high level of satisfaction, those findings did not include users who did not participate in evaluation, which could represent subjects less adhered to the treatment. Results highlighted the need of periodical follow-ups to ensure the effectiveness of auditory rehabilitation.

## INTRODUCTION

Hearing aids are one of the therapeutic options available for hearing loss. In Brazil, they can be acquired in private hearing aid dispensers or in publicly-funded hearing care services accredited by the Brazilian Public Health Care System (*“Sistema Único de Saúde”*, or SUS).

Long-term use and satisfaction with hearing aids can be used to analyze the effectiveness of hearing rehabilitation1., 2., 3.. Hearing aid use can be evaluated through data logging or through interview with the hearing aid user. Satisfaction with hearing aids can be examined through self-report questionnaires, such as Satisfaction with Amplification in Daily Life questionnaire, or SADL™ ^3^.

Several studies pointed a tendency of reduction in hearing aid use throughout time4., 5., 6., 7.. Most of problems regarding reduction in hearing aid use can be solved with interventions and fittings in a long-term follow-up program, even after fitting process. However, considering the clinical practice of the publicly-funded services analyzed, these follow-up appointments don't occur effectively6., 8., 9..

Few studies have looked into long-term use and satisfaction in Brazilian hearing aid users, or even long-term effectiveness of auditory rehabilitation in Brazil^10^.

The objective of this study was to evaluate the use of hearing aids dispensed by a publicly-funded health service, as well as the interventions needed and users' satisfaction.

## METHOD

This was a prospective observational cross-sectional study, approved by protocol number 0160/10 from Ethics Committee of Research Projects Analysis.

Users whose hearing aids were dispensed one year ago by a publicly-funded health service were selected from medical records and invited to participate in this study by telephone. The inclusion criteria were: adults (at least 18 years old) with mild to moderately severe, bilateral and symmetric sensorineural hearing loss, who wore digital behind-the-ear hearing aids with nonlinear amplification in both ears for one year, with no previous experience with hearing aids. Subjects with neurologic disorders, outer or middle ear malformations, manual dexterity problems or fluctuating hearing loss were the exclusion criteria from study sample.

### Procedures

All participants signed an informed consent form and underwent an evaluation consisted of six steps, described as follows:

Step 1: Initial interview

Participants answered open-set questions regarding hearing aid use:

a. “Do you use your hearing aids? Do you have any problem?”
b. “Do you usually wear both hearing aids (bilateral), only one (unilateral) or none of them?”
c. “What is the main reason for not wearing your hearing aids?”
d. “Approximately how many hours per day do you use your hearing aids?”
e. “Have you made some kind of follow-up (private hearing care center or hearing care services of the public health care system - SUS) during this year using your hearing aids?”

Step 2: SADL™ questionnaire

Each subject answered the SADL™ items as an interview with the researcher, which allowed explanations about the doubts according to their difficulties. Considering that all subjects had hearing aids provided by public resources and not purchased, item 14 (“The cost of the hearing aids seems reasonable?”) was used in order to assess satisfaction regarding costs of batteries and maintenance of hearing aids, unlike the original proposal^3^.

Step 3: Visual inspection of external auditory canal, to verify the presence of earwax blocking the ear canal.

Step 4: Pure tone audiometry, to assess possible changes in hearing thresholds that could influence hearing aid fitting.

Step 5: Earmold check, to analyze integrity and quality and factors that may cause physical discomfort.

Step 6: Hearing aid's sound check with stethoscope, to verify quality of amplification.

All orientations and suggested interventions were registered and classified as major or minor^6^ after each assessment.

Data were analyzed using descriptive statistics.

## RESULTS

Although 376 subjects were initially selected as sample of this study, active search by telephone resulted in 200 participants who attended the evaluation appointments (47% of sample loss).

Considering all 200 hearing aids users assessed in this study, 102 (51%) were female and 98 (49%) were male, mean age of 71.3 years. Major education level was uncompleted elementary school (58.5%) and 55.5% were retired.

Only 68% of subjects returned for follow-up appointments in the first year, even though all of them were oriented about the importance of follow-ups during fitting process.

### Descriptive analysis of hearing aids use after one year

The analysis of hearing aids use considered three aspects: mode of hearing aid use (bilateral, unilateral or none), reasons for non-use of hearing aids and hours using hearing aids.

Table 1 shows the results for mode of hearing aid use after one year.

**Table 1:** Frequency and percentage frequency distribution of mode of hearing aid use.

Mode of hearing aid use	N	%
Bilateral	153	76.5
Unilateral	21	10.5
None	26	13
Total	200	100

The analysis of reasons for non-use of hearing aids regarding users who were not using one or none of them is at Table 2.

**Table 2:** Frequency and percentage frequency distribution of reasons for non-use of hearing aids.

Reasons for non-use of hearing aids	N	%
Sound discomfort	12	25.5
Manual dexterity	7	14.9
Poor benefit	6	12.8
Health problems	4	8.5
Fit and comfort	3	6.4
Faulty hearing aid	5	10.7
Problems with earmold	2	4.3
Lost hearing aid	2	4.3
Couldn't buy more batteries	2	4.3
Itch	1	2.1
Don't accept use	1	2.1
Don't need to use both hearing aids	1	2.1
Occlusion effect	1	2.1
Total	47	100

Regarding to hours of use, the average was 8.6 hours per day, minimum of 0 hours per day and maximum of 18 hours per day; 60.5% reported to wear their hearing aids for eight hours or more and 13% were not wearing them at all.

### Descriptive analysis of necessary interventions after one year

Table 3 shows data related to the interventions^6^ needed by hearing aid users after one year.

**Table 3:** Frequency and percentage frequency distribution of major and minor interventions necessary to hearing aid users.

Necessary interventions	N	%
MAJOR		
Alteration of more than 5 dB in hearing aid gain	52	26%
Refer to ear nose and throat doctor	29	14.5%
Repair of hearing aid	14	7%
Change of hearing aid	0	0%
MINOR		
Explanation on phone use	63	31.5%
Explanation on maintenance and cleaning	56	28%
Alteration of 5 dB or less in hearing aid gain	45	22.5%
Counseling about benefits and limitations of hearing aids	45	22.5%
Explanation on earmold insertion	35	17.5%
Earmold alteration	27	13.5%
Explanation on draining and purchase of batteries	23	11.5%
Explanation on programme use	17	8.5%
Advice on benefits of using both hearing aids	15	7.5%
Earmold retube	11	5.5%
Changeof earmold	9	4.5%
Change on programs	9	4.5%
Explanation on earmold or hearing aid insertion	7	3.5%
Explanation of the need for regular earmold retubing	4	2%
Counseling for tinnitus treatment	4	2%

Major interventions^6^ were necessary for 81 hearing aid users and 118 needed only minor interventions^6^. Only one subject did not need any kind of intervention.

### Descriptive analysis of satisfaction with hearing aids using the SADL™ questionnaire

Users who kept wearing their hearing aids bilaterally or unilaterally answered SADL™ questionnaire. Results were analyzed considering Global Satisfaction and subscales: Positive Effects, Negative Features, Service and Costs and Personal Image, as seen in Table 4.

**Table 4:** Descriptive statistics for global satisfaction and subscales of SADL™ questionnaire.

Subescale	N	Mean	Standardd eviation	Minimum	Median	Maximum
Positive effects	174	5.8	1.2	2.0	6.0	7.0
Negativ efeatures	174	6.2	1.1	3.0	6.6	7.0
Service and costs	174	6.3	0.8	4.3	6.3	7.0
Personal image	174	6.2	0.8	3.0	6.3	7.0
Global satisfaction	174	6.0	0.8	3.8	6.2	7.0

## DISCUSSION

This study had a descriptive and exploratory design and intended to be a starting point for a discussion about the effectiveness of Brazil's public policies on hearing health, and based in its findings, originate new conclusive studies regarding hearing aid use including users from other places of the country.

Initially, 376 subjects were selected for this study according to inclusion and exclusion criteria. However, there was a considerable loss of the sample in active search by telephone, and only 53% of the sample primarily chosen attended the evaluation appointment, totalizing 200 subjects. The reasons for sample loss were: failure to contact by telephone, unattended appointments, failure to schedule appointments by health problems or difficulty to go to publicly-funded health service, returned hearing aids and death. These results will be discussed in an upcoming study, but show that hearing aid users have difficulties to attend follow-up appointments in this publicly-funded health service, which affects long-term effectiveness of auditory rehabilitation.

As an important measure to manage and improve the quality of publicly-funded health services and the effectiveness of auditory rehabilitation in Brazil, more studies should be executed in other services in different parts of the country, taking into consideration the existing guidelines1., 2., 10., 11., 12. and, criteria and findings of this initial study, which was conducted in a publicly-funded health service that is considered a national benchmark for this type of care.

### Hearing aid use after one year

Although hearing aid non-use rates vary in literature, the rate of 13% of non-use observed in the present study is higher than 3% seen in an Australian study^13^, 3.1% from Switzerland^14^ and 12.4% from United States^7^. On the other hand, it is lower than 57% from a British study^8^, 25% from Finland^15^ and 32.26% seen in hearing aid users from other state of Brazil^9^. The different criteria used for sample selection of these studies, such as, age groups, period using hearing aids, form of acquisition (purchased, partially or totally funded hearing aids), may have contributed to the variety of non-use rates seen in literature. Further investigations about this topic should use standardized criteria to improve data comparability between different studies.

As seen in Table 1, most of users from this study kept using hearing aids bilaterally, in accordance with recommendations given during fitting process1., 2..

In cases of unilateral or no use of hearing aids, the subjects were interviewed about the reason to abandon use, choosing only one main argument. As noted in Table 2, the main reasons stated were: “sound discomfort” (25.5%), “manual dexterity” (14.9%) and “poor benefit” (12.8%), among others. The problems stated by the users could be solved with effective periodical follow-ups, with adjustments in gain and/or device programming, physical adjustments, electroacoustical circuit inspection and cleaning, and by use instructions and advising1., 2., 6., 8.. These problems differ from literature findings regarding most recurrent reasons for non-use of hearing aids, which were: poor hearing benefit, non-acceptance of hearing loss or refusal to use a hearing aid, sound or physical discomfort, unattended expectations, stigma or aesthetic factors and financial restrictions4., 7., 8., 9., 14., 16., 17., 18., 19., 20.. Most of the problems indicated by literature are not due to necessity of adjustments or repair of hearing aids, but to subjective factors to users, which depend on the users themselves to be solved.

This fact suggests the necessity to improve long-term follow-up system of publicly-funded health services and users' access to follow-ups appointments, since 32% did not make any kind of follow-up in the first year of hearing aid use, differently from what is proposed in guidelines for audiology services1., 2., 21., 22.. The lack of orientation and/or necessary interventions tend to cause reduction in hearing aid use, benefit and satisfaction over time4., 6., 23., 24.. Thereby, it is reasonable to question if necessities of Brazilian hearing impaired people continue to be matched over time (not only during fitting process), as well as the quality and effectiveness of the assistance offered by Brazilian publicly-funded audiology services10., 25., 26., 27., 28..

In relation to hours of hearing aid use, most of users reported to use hearing aids for at least 8 hours daily and the average of use was 8.6 hours, which suggests hearing aids use during most of daily activities.

The average of use seen in this study is lower than the daily average of 9.5 hours found in United States^7^.

### Necessary interventions after one year

Practically all users of this study (99.5%) needed some kind of intervention after one year, and 40.5% had substantial problems with their hearing aids requiring major interventions. This rate was similar to the one found in U.K.^6^, in which 39% of hearing aid users needed major interventions after a reassessment appointment, yet they were subjects who had not made any kind of follow-up during three years of hearing aid use.

According to Table 3, the most recurrent interventions needed were different from U.K. study^6^ and consisted basically of orientation and counseling procedures, such as “explanation on phone use” (31.5%), “explanation on maintenance and cleaning” (28%) or “counseling about benefits and limitations of hearing aids” (22.5%). Also there was necessity of interventions for problems due to long term use, like “alteration of more than 5 dB in hearing aid gain” (26%) and “earmold retubing” (5.5%).

It is also important to highlight the amount of users that needed “explanation on draining and purchase of batteries” (11.5%), who did not know about the necessity of changing batteries or where to buy them, even after one year of hearing aid use. Some subjects were not using effectively their hearing aids because they did not have financial resources to buy batteries, so they did not wear any hearing aid for some period or used them for few hours to save battery charge.

Some users of this study still kept on wearing their hearing aids even with substantial problems for use, like faulty earmolds, faulty hearing aids or drained battery. Many of them were not aware of the necessity of adjustments and maintenance over time, for many “it was enough to use hearing aids just the way they were given”, according some users of this study. This behavior was also seen in other Brazilian hearing aid users^9^ and was different from the conception of users from other countries6., 14..

Based on the facts seen in this study, many Brazilian users still have doubts and difficulties in dealing with their hearing aids, even with orientations received during fitting process. Considering that most of users are elderly people, with uncompleted elementary school and poor speech comprehension due to hearing loss, probably the time granted for orientation and counseling is not enough to elucidate all questions they have, which damages use, benefit, satisfaction and even adherence to hearing rehabilitation, as well as its effectiveness over time16., 17., 19., 20..

### Satisfaction with hearing aids using SADL™ questionnaire

According to SADL™ analysis, 97.49% of hearing aids users were satisfied and global satisfaction mean score was 6.0 (Table 4), corresponding to “very satisfied” with hearing aids^3^. It was the highest mean score compared to others from literature, higher than 5.27 of Australian hearing aid users^13^, 4.87 of American users^29^, 5.28 of elderly users from the state of Tocantins (Brazil)^30^, 5.55% from other Brazilian study^31^ and 5.77% of Brazilian users with profound or severe hearing loss^32^.

All scores from SADL™ subscales also represented high level of satisfaction with hearing aids^3^ within the users. “Service and costs” subscale had the highest score (6.3) and “Positive effects” the lowest (5.7%), even though all subscales also had scores within “very satisfied”^3^.

The application of question 14 from SADL™ differently from the original^3^ was an effective approach to verify satisfaction with financial costs associated to hearing aid use. Subjects with financial difficulties could reveal they were not using their hearing aids because they did not have resources to purchase batteries or pay the repair costs.

SADL™ questionnaire was chosen for containing direct questions, being focused on clinical application and it has been used to evaluate satisfaction in Brazilian hearing aid users from other publicly-funded audiology services30., 31., 32., 33., 34.. However, despite the high level of satisfaction observed among the participants of this study, it is questionable how well this instrument can evaluate hearing aid satisfaction of users with this profile, because most of them needed explanations from the researcher to be able to answer the questions. Alterations in questions involved language simplification, use of practical examples, graphics and illustrations. Besides, many users showed an attitude of “gratitude” for all the assistance received and gave overestimated answers in questionnaire, which did not match the reality, behavior also seen in other Brazilian study^10^.

## CONCLUSION

• Active search by telephone showed that hearing aid users who attend a publicly-funded health service have difficulties to attend follow-up appointments.
• Most of the users that came for evaluation kept using hearing aids bilaterally (76.5%); non-use rate was 13%.
• Practically all users (99.5%) needed some kind of intervention, which emphasize the necessity of periodical follow-ups to ensure long-term effectiveness of hearing rehabilitation.
• Considering users who came for evaluation and kept wearing their hearing aids, most of them (98%) were satisfied with amplification according to SADL™ questionnaire. However, this result did not include users who did not come to evaluation appointment, which could represent subjects less adhered to the treatment.
